# Phenyl 1,2,3-Triazole-Thymidine Ligands Stabilize G-Quadruplex DNA, Inhibit DNA Synthesis and Potentially Reduce Tumor Cell Proliferation over 3′-Azido Deoxythymidine

**DOI:** 10.1371/journal.pone.0070798

**Published:** 2013-08-19

**Authors:** Jerald Mahesh Kumar, Mohammed M. Idris, Gunda Srinivas, Pallerla Vinay Kumar, Vuppalapaty Meghah, Mitta Kavitha, Chada Raji Reddy, Prathama S. Mainkar, Biswajit Pal, Srivari Chandrasekar, Narayana Nagesh

**Affiliations:** 1 CSIR-Centre for Cellular and Molecular Biology, Hyderabad, India; 2 CSIR-Indian Institute of Chemical Technology, Hyderabad, India; Medical College of Wisconsin, United States of America

## Abstract

Triazoles are known for their non-toxicity, higher stability and therapeutic activity. Few nucleoside (**L1**, **L2** and **L3**) and non-nucleoside 1,2,3-triazoles (**L4–L14**) were synthesised using *click chemistry* and they were screened for tumor cell cytotoxicity and proliferation. Among these triazole ligands studied, nucleoside ligands exhibited higher potential than non-nucleoside ligands. The nucleoside triazole analogues, 3′-Phenyl-1,2,3- triazole-thymidine (**L2**) and 3′-4-Chlorophenyl-1,2,3-triazole-thymidine (**L3**), demonstrated higher cytotoxicity in tumor cells than in normal cells. The IC_50_ value for **L3** was lowest (50 µM) among the ligands studied. **L3** terminated cell cycle at S, G2/M phases and enhanced sub-G1 populations, manifesting induction of apoptosis in tumor cells. Confocal studies indicated that nucleoside triazole ligands (**L2/L3**) cause higher DNA fragmentation than other ligands. Preclinical experiments with tumor-induced mice showed greater reduction in tumor size with **L3**. *In vitro* DNA synthesis reaction with **L3** exhibited higher DNA synthesis inhibition with quadruplex forming DNA (QF DNA) than non quadruplex forming DNA (NQF DNA). T_m_ of quadruplex DNA increased in the presence of **L3**, indicating its ability to enhance stability of quadruplex DNA at elevated temperature and the results indicate that it had higher affinity towards quadruplex DNA than the other forms of DNA (like dsDNA and ssDNA). From western blot experiment, it was noticed that telomerase expression levels in the tissues of tumor-induced mice were found to be reduced on **L3** treatment. Microcalorimetry results emphasise that two nucleoside triazole ligands (**L2**/**L3**) interact with quadruplex DNA with significantly higher affinity (K_d_≈10^−7^ M). Interestingly the addition of an electronegative moiety to the phenyl group of **L2** enhanced its anti-proliferative activity. Though IC_50_ values are not significantly low with **L3**, the studies on series of synthetic 1,2,3-triazole ligands are useful for improving and building potential pro-apoptotic ligands.

## Introduction

Drugs used for the control of cancer are broadly classified into two groups, cytotoxic (cell killing) and cytostatic (cell stabilizing). Several nitrogen-containing ligands have been successfully tested as cytotoxic drugs for cancer treatment. Among them, triazole derivatives were found to be ideal because they are non-toxic, more likely to be water soluble and highly stable [1]. They are not naturally occurring scaffolds but their roles in analgesic [2], anti-inflammatory [3], [4], antiviral [5], antimicrobial [6], [7], antifungal [8]–[10], antibacterial [11], antitubercular [12] and antitumor [13] activities are well documented. Further, several triazole derivatives were tested for their anticancer activity [14]. Considering the broad spectrum of activities that triazoles exhibit, 2 different classes of triazole scaffolds (with and without nucleoside) were synthesized and their physico-chemical, biological characteristics as well as their efficacy in reducing tumor size and control tumor cell proliferation was examined.

During 1960s, Zidovudine (AZT), an azido derivative, was designed to use in chemotherapy for leukaemia [15]. But its use as an antitumor drug was discontinued as it has failed to act specifically on tumor cells. Further, azides are known for their instability, explosive and toxic nature [16]–[18]. Therefore, there is an ongoing search for ideal molecules that reduce tumor cell proliferation and the azido moiety was converted to more stable, safe and non-toxic triazole scaffold. Earlier, similar or same molecules were successfully tested as inhibitors for reverse transcriptase, *Mycobacterium tuberculosis* thymidine monophosphate kinase (TMPKmt) and human mitochondrial thymidine kinase 2 (TK-2) [19]–[22]. Nitrogen-containing ligands like TMPyP4, BRACO-19, Telomestatin etc., were found to be efficient in stabilizing quadruplex DNA and controlling tumor cell proliferation [23]–[27]. We have earlier reported the interaction and stabilization of quadruplex DNA by several nitrogen-containing ligands [28]–[31].

To the best of our knowledge, we are the first group to demonstrate the potential of Phenyl-1,2,3-triazole nucleosides such as 3′-Phenyl-1,2,3-triazole-thymidine (**L2)** and 3′-4-Chlorophenyl-1,2,3-triazole-thymidine (**L3**) in controlling telomerase expression levels in the tissues of tumor-induced mice, specifically stabilize telomere G-quadruplex DNA and reduce tumor cell proliferation. Recently, it was reported that 1,2,3-triazole ligands selectively bind and stabilize quadruplex DNA [32]–[34]. A mixed pyrimidine moiety having phenyl-1,2,3-triazole when included in a synthetic oligonucleotide, enhanced stability of DNA by π-π stacking of phenyl-1,2,3-triazole moiety in the major groove [35]. Recent studies indicate that quadruplex DNA stabilizing ligands have inherent ability to control telomerase and function as anti-proliferative ligands [36].

Quadruplexes are unique, non-canonical DNA structures, found in the human genome, preferably in telomeres and regions slightly upstream of the oncogene promoter. In human somatic cells, telomeres have about 500–3000 [TTAGGG] repeats, which get shortened with age. Recently, it was estimated that the human genome contains about 300000 sequences potentially capable of forming quadruplex structures [37]. G-quadruplex structures play a crucial role in regulating transcription, and therefore, have influence on cellular activities. Stabilization of G-quadruplex structure was reported to control gene expression and subsequently control oncoprotein expression in tumor cells [38].

In an attempt to improve the efficacy of synthetic azido ligand in controlling tumor cell proliferation, under less toxic conditions, an array of 1,2,3-triazole molecules were synthesised and their potentials were studied using various *in vivo* and *in vitro* experiments. We have shown that the addition of an electronegative group to the phenyl group enhance the potential of phenyl-1,2,3-triazole-thymidine ligand.

## Materials and Methods

### Human cell lines

Normal human cell lines CRL-2115 and CRL-2120 were obtained from American Type Culture Collection (Rockville, MD). Human tumor cell lines NCM-460, He La, MCF-7, B16F10 and A549 were obtained from National Centre for Cell Sciences, India and Lonza Cologne GmbH (Germany). The cell lines were grown according to the supplier's recommendation.

### DNA and triazole ligand synthesis

Human quadruplex forming DNA [HQF DNA- 5′- TTAGGGTTAGGGTTAGGGTTAGGG-3′ or d(TTAGGG)_4_] from telomere region, quadru- plex forming DNA (QF DNA) and non-quadruplex forming DNA (NQF DNA) was synthesized by β-cyanoethyl phosphoramidite chemistry and purified by HPLC. Oligonucleotide stock solutions were prepared by dissolving them in BPES buffer solution (30 mM potassium phosphate, pH 7.0, with 100 mM KCl). Oligonucleotide solutions were extensively dialyzed against the BPES buffer using a 1000 Da molecular weight cut off membrane at 4°C. The final concentration was determined by UV absorbance at 254 nm with molar extinction coefficient determined by using the nearest neighbour calculation for single stranded DNA [39] and the absorbance of thermally denatured constructs extrapolated back to 25°C by total phosphate analysis technique [40]. Extinction coefficient of HQF DNA (ε_254_), QF DNA (ε_260_) and NQF DNA (ε_260_) was found to be 254064 M^−1^ cm^−1^, 484500 M^−1^ cm^−1^ and 219100 M^−1^ cm^−1^ respectively. The dsDNA (poly-dA-dT and poly-dT-dA) and ssDNA (poly-dT) were purchased from Sigma Aldrich (USA).

**Figure 1:**
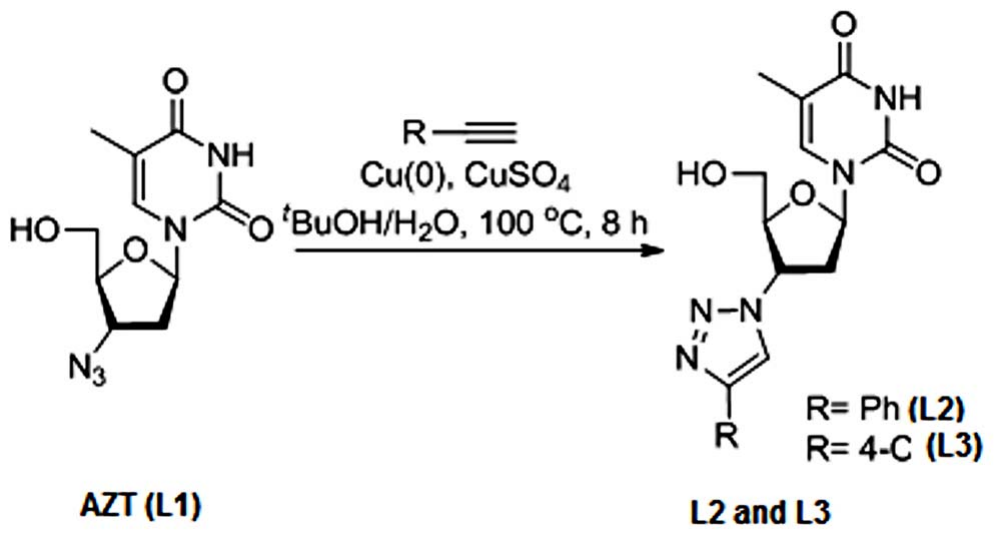
Synthesis of L2 and L3 from L1.

**Figure 2:**
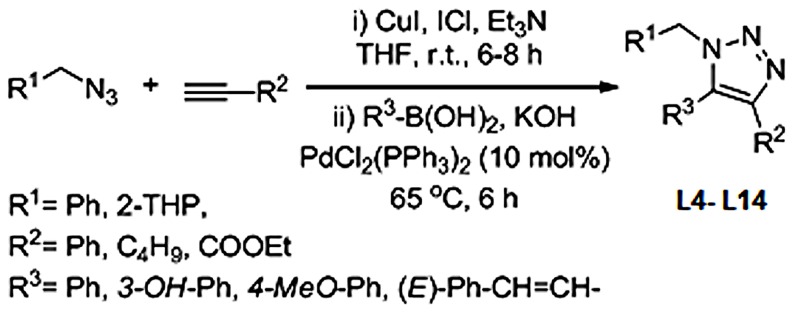
One-pot synthesis of L4–L14.

All the ligands except, 1-(4-azido-5-(hydroxylmethyl)tetrahydrofuran-2-yl)-5-methyl- pyrimidine-2,4-(*1H*,*3H*)-dione, popularly known as 3′-Azido-3′-deoxythymidine (**L1**), were synthesized using *‘click chemistry’*. **L1**, a well known, commercially available, telomerase inhibitor, was used as source material for the synthesis of **L2** and **L3**. Among triazole scaffolds, 1-(5-(hydroxyl-methyl)-4-(4-phenyl-*1H*-1,2,3-triazol-1-yl)tetrahydrofuran-2-yl)-5-methylpyrimidine-2,4(*1H*,*3H*)-dione, referred in this study as 3′-Phenyl-1,2,3-triazolo-thymidine (**L2**) and 1-(4-(4-(4-chlorophenly)-1H-1,2,3-triazole-1-yl)-5(hydroxyl-methyl)tetra -hydrofuran-2-yl)-5-methylpyrimidine-2,3(1H,3H)-dione, referred as 3′-4-Chloro-phenyl-1,2, 3-triazolo-thymidine (**L3**), were synthesized using the method reported by Poecke *et al* [22]. The stock solutions of all the ligands used in the present study were prepared in 1∶1 water∶methanol. **L2** and **L3** ligands differ only with respect to the pharmacophores attached to 3′ of thymidine. The remaining non-nucleoside triazole scaffolds differ from each other in the pharmacophores attached to the 1^st^, 4^th^, 5^th^ positions (-R1, -R2 and –R3) of 1,2,3-triazole ring. Synthesis details of nucleoside 1,2,3-triazole ligands (**L2** and **L3**) and non-nucleoside 1,2,3-triazole ligands (**L4**–**L14**) are documented in the Figure 1 and 2 respectively.

### MTT assay

Normal human cell lines (CRL-2115, CRL-2120) as well as human cancer cell lines (NCM-460, He La, MCF-7, B16F10 and A549) were cultured according to the supplier's recommendations. To study the cytotoxicity of the ligands, after 80% confluence, cells were trypsinized with 0.1% trypsin-EDTA and harvested by centrifugation at 500× g. Serial dilutions of cells were made from 1×10^6^ to 1×10^3^ cells per ml. The cells were seeded in triplicate in a 96 well plate. The suspended cells were treated with 50 µM, 100 µM, 150 µM, 200 µM, 250 µM and 300 µM of each ligand, at two different time points (24 h and 48 h). Normal as well as tumor cells to which no ligand was added served as control. After incubation, MTT (100 µl, 5 mg mL^−^1) solution was added to each well and the cell viability was determined by measuring the ability of cells to transform MTT to a purple coloured formazan dye. The absorbance of samples at 570 nm was measured using a UV-Visible spectrophotometer. Percentage of viable cells was calculated using the formula given below.
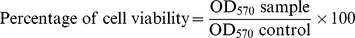
Where the OD_570_ (sample) corresponds to absorbance obtained from the wells treated with ligands and the OD_570_ (control) represents the absorbance from the wells in which no ligand was added.

### Cell count assay

Normal and tumor cell lines were seeded at 20000–40000 cells/cm^2^ with 0.2 mL/cm^2^ media and incubated for 48 h at 37°C, 6% CO_2_, 95% relative humidity. Cells were treated with 200 µM, 125 µM and 50 µM of **L1, L2, L3** respectively (at their respective IC_50_ value). After 24 h and 48 h of incubation, cells were washed with 300 µl PBS or 0.02% EDTA, trypsinized, followed by harvesting at 37°C. 200 µl cell suspension was transferred into 1.5 ml microcentrifuge tube. To the cell suspension, 300 µl PBS followed by 500 µl of 0.4% trypan blue solution was added. To get the homogenised solution, the microcentrifuge tube was gently tapped for 5 min, 20 µl of cell suspension was loaded in to a chamber of the haemocytometer.

### Flow cytometry, Confocal microscopy studies

Since MTT and cell culture assays yielded encouraging results with B16F10 cells they were used for confocal and flow cytometry experiments. After 90% confluence, the cells were treated with 200 µM, 125 µM and 50 µM of **L1, L2, L3** respectively, for 3 h. 1×10^6^ cells were washed with 2× binding buffer and resuspended in 100 µl binding buffer and Annexin-V-FITC (1.0 µg). The cells were then incubated at room temperature for 10 min, followed by the addition of 400 µl of binding buffer containing 1 µl of Propidium Iodide (PI). Stained cells were analyzed using FACS Calibur Flow Cytometer from B.D. Biosceinces (USA). Annexin-V-FITC and PI labelled cells were excited using 488 nm solid-state laser and fluorescence emission intensity was captured using 530/30 and 585/42 band pass filters respectively.

For confocal microscopic studies, B16F10 cells were rinsed with 1× PBS and incubated with 2.5% formaldehyde in PBS for 10 min at room temperature, followed by permeabilization with 0.5% Triton X-100 for 5 min. The fixed cells were incubated with 10% FCS in PBS for 1 h at room temperature. The culture plate was then washed with 1× PBS and mounted in mounting medium (Vectashield), containing anti-fade reagent with 300 nM DAPI and incubated for 1–5 min. The cells were rinsed with PBS several times and viewed using a fluorescent microscope.

### Stop assay

For this assay, synthetic oligonucleotides rich in guanine that have potential to form G-quadruplex DNA structure (QF-template and QF-primer) as well as oligonucleotides with less guanines, which do not form G-quadruplex DNA structure (NQF-template and NQF-primer) were considered. Sequences of the oligonucleotides used are shown in Table 1. The G-quadruplex structure forming region of QF oligonucleotide is shown in bold letters.

**Table 1:** Sequences of QF and NQF oligonucleotides used in stop assay.

S.No	Oligonucleotide name	Oligonucleotide Sequence
1	QF DNA Primer	5′-ACGACTCACTATAGCAATTGCG-3′
2	QF DNA Template	5′**AGGGTTAGGGTTAGGGTTAGGGG**CCACCGCAATTGCTATAGTGAGTCGT-3′
3	NQF DNA Primer	5′-AATGAA-3′
4	NQF DNA Template	5′-ACTTACTTACT TACTTACTTACTTACTTACTTACTT-3′

To the PCR reaction mixture (25 µl) containing 10 mM Tris-HCl, pH 8.3, with 50 mM KCl, 1.5 mM Mg(OAc)_2_, 5 µM of each oligonucleotide, 1 mM dNTPs, 5 units of Taq polymerase, **L1**, **L2**, **L3** were added at various concentrations (50 µM, 100 µM, 150 µM, 200 µM), and PCR reactions were performed. PCR mixture without any ligand was considered as control. The primers were 5′ end-labelled with [γ-^32^P] ATP using 1 µl of polynucleotide kinase and 5 µl of 10× kinase buffer and the contents were incubated at 37°C for 5 min. Stop assay was performed using an ABI thermal cycler. Initial denaturation was done at 94°C for 3 min, followed by 20 repeated cycles each having 94°C for 30 sec, 58°C for 30 sec, and 72°C for 30 sec. After PCR, 5 µl of the amplified product was loaded on a 12% non-denaturing polyacrylamide gel with 1×TBE. The *in vitro* DNA synthetic band intensity was measured using Bio Rad Gel Doc XR+system (USA). The band intensity of the PCR product obtained with no ligand was taken as control for all the quadruplex forming DNA samples (QF DNA). Percentage of DNA synthesis inhibition was calculated using equation 1, whereas, inhibition constant, K_i_ (using Cheng-Prusoff equation) for each ligand was calculated by using equation 2.
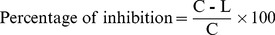
(1)


C - Band intensity with control.

L - Band intensity with ligand.
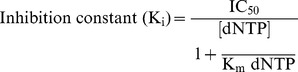
(2)


IC_50_-Half maximal inhibitory concentration.

[dNTP] – Concentration of dNTPs used.

K_m_ - Michaelis constant.

### Preclinical studies

C57BL6/J mice were used from the animal house facility located at CCMB (Hyderabad, India). All animal experimental studies were carried out in strict accordance with the recommendations in the guide for the care and use of laboratory animals of Centre for Cellular and Molecular Biology (CCMB). All the animal experimental protocols were approved by the Institutional Animal Ethical Committee (IAEC) of Centre for Cellular and Molecular Biology (CCMB with Permit number: CPCSEA 20/1999, Ministry of Environment and Forest, Government of India).The protocol was approved by the Institutional Animal Ethical Committee (IAEC) of CCMB (Protocol number: IAEC 16/2012). All the surgery was performed under sodium pentobarbital anaesthesia, and all efforts were made to minimize suffering. C57BL6/J mice, 6–8 weeks old, were subcutaneously injected with 2×10^5^ B16F10 melanoma cells, in the intraperitoneal region. After the tumor attained a size of 500–1000 mm^3^ (≈9 days following cancer cell implantation), mice were grouped and treated as follows.

Group (i) was treated with vehicle control (4 mice), group (ii) with 200 µM of **L1** (4 mice), group (iii) with 125 µM of **L2** (4 mice) and group (iv) with 50 µM of **L3** (4 mice). These experiments were repeated thrice and mean values were considered. Size of tumor was measured every day at a fixed time. The tumor sizes were calculated using the formula (0.5 ab^2^), where ‘a’ was the largest dimension and ‘b’ was the smallest dimension of the tumor. The experiment was terminated when the average tumor volume of the control/treated group was 10000–12000 mm^3^. To validate the effect of ligands on mice survival, in the same manner, three groups of mice were taken and 200 µM, 125 µM and 50 µM of **L1**, **L2** and **L3** were injected to each mouse on the alternate days for 2 weeks and their activity was observed, till they die naturally.

### Western blotting

Western blot analysis was carried out to study the expression of telomerase in ligand treated as well as untreated tissues of tumor-induced mice. Total proteins were extracted from the tumor tissues into solubilisation buffer (7 M urea, 2 M thiourea, 4% CHAPS, 18 mM Tris–HCl, 14 mM Trizma base, 2 tablets EDTA protease inhibitor, 0.2% Triton-X, 50 mM DTT) following homogenization and sonication. Extracted proteins were quantified against BSA standard using an Amido black assay method [41]. 40 µg of total protein obtained from different time points (48 h, 72 h and 216 h) were electrophoresed on a 10% SDS-PAGE and transferred onto a PVDF membrane using wet-transfer method. Immunoblot analysis of the telomerase reverse transcriptase (TERT) expression was performed using anti-TERT antibody (Pierce, Thermo Scientific) as primary antibody and anti-rabbit HP conjugated secondary antibodies (Pierce, Thermo Scientific). The immuno- blots were scanned using the ECL detection method to estimate the telomerase expression levels in the tissues of tumor-induced mice. The endogenous β-actin expression level was considered as control.

### Binding and interaction studies

To further corroborate the efficiency of various synthetic ligands in interacting and stabilizing human telomeric quadruplex DNA, melting studies of DNA with and without ligands was carried out following SYBR Green fluorescence as reported earlier [42], using Eppendorf realplex^4^ mastercycler (Hamburg, Germany). SYBR Green (SG) was reported to intercalate to quadruplex DNA and enhance fluorescence [43]. When quadruplex DNA melts to form ssDNA structure, SG fluorescence intensity gradually reduces as SG has no binding to ssDNA. Each time, 5 µM of HQF DNA [d(T_2_AG_3_)_4_)], 5 µM of dsDNA (prepared by mixing equal concentration of poly-dA-dT and poly-dT-dA), 5 µM ssDNA ( poly dT alone) and 1 µM of each ligand was incubated at 4°C for 16 h, and used for melting studies. For melting studies, 1 µM of ligands was considered instead of 200 µM, 125 µM, 50 µM of **L1**, **L2** and **L3** respectively, because the aim of the experiment is to find the extent of stability provided to quadruplex DNA by each ligand at low concentration. Affinity, stoichiometry and thermodynamic parameters for quadruplex-ligand complex were studied with 60 µM of ligand and 10 µM of HQF DNA using Microcal VP-ITC (Northampton, MA, USA). Ligand addition to G-quadruplex DNA was made every 180 seconds. Titration experiments were performed thrice and blank correction was made each time to minimize error.

### Statistical Analysis

The results obtained from different experiments were subjected to statistical analysis. SPSS (Version 18.0, Chicago, IL) was used for all the statistical analysis. The statistical parameters like mean value and standard deviation were calculated using descriptive statistics. Data was considered significant if p value≤0.05. The set of data with p values≤0.001, 0.01 and 0.05 were marked with symbols *, # and **/no symbol, respectively in each graphical representation.

## Results and Discussion

### MTT assay

**Figure 3:**
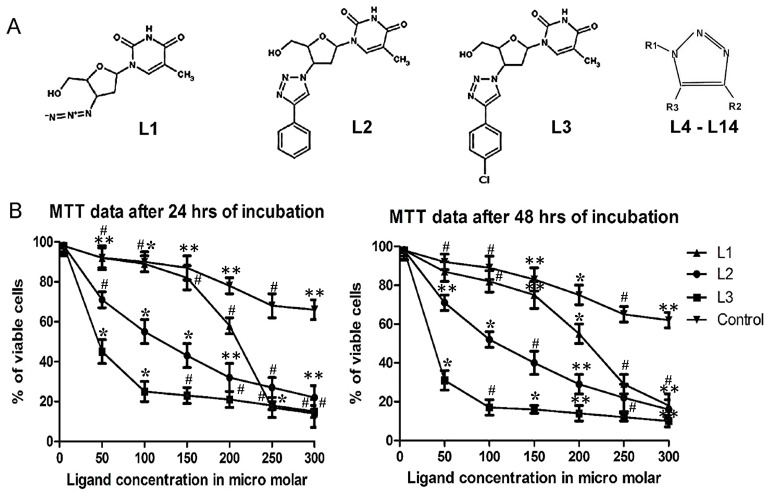
Structure of ligands used and the MTT assay. **A.** Structure of nucleoside 1,2,3-triazole ligands **L1, L2, L3** and the basic structure of non-nucleoside 1,2,3-triazole ligands (**L4–L14**). The position of various pharmacophores on ligands (**L4–L14**) are labelled with -R1, -R2 and -R3. **B.** MTT assay with B16F10 cells on treatment with 50 µM, 100 µM, 150 µM, 200 µM, 250 µM and 300 µM concentration of **L1**, **L2** and **L3** for 24 h and 48 h. The set of data with reducing order of significance (p value≤0.001, 0.01 and 0.05) were marked with *, # and ** respectively.

This assay indicates the rate of tumor cell proliferation and useful to assess the potential of synthetic ligands in the induction of apoptosis. MTT assay was performed by following the protocol described by Wilson *et al* [44]. Structure of synthetic ligands used for MTT assay was shown in Figure 3A. From the assay it is clear that among the ligands used in the present study, nucleoside ligands exhibited lower half maximal inhibitory concentration (IC_50_) than the non-nucleotide ligands. Figure 3B indicates that the half maximal inhibitory concentration (IC_50_) for nucleoside ligands (**L1**, **L2** and **L3**) with B16F10 cells were around 200 µM, 125 µM and 50 µM respectively. Whereas cells not treated with any ligand (control cells) continue to proliferate without any inhibition. Though the cytotoxicity of **L3** ligand (50 µM), is not very significant, a comparative study of various nucleoside and non-nucleoside triazole molecules will provide insights into the role of pharmacophores attached, effect of size and structure of 1,2,3-triazole ligands on the anti-proliferative activity as well as for building suitable triazole molecules for potential antitumor activity. Triazoles are known for safety and therapeutic applications for a prolonged period. The drugs having triazole scaffold like Vorozole, Letrozole and Anastrozole are under use for cancer treatment for several years [45]. From MTT assay it is evident that among nucleoside ligands, 1,2,3-triazole ligands (**L2/L3**) are comparatively more efficient than azido thymidine (**L1**) and non-nucleoside triazole ligands (**L4–L14**) in bringing down tumor cell proliferation. Hence more emphasis is laid on the relatively new and higher potential, nucleoside 1,2,3-triazole ligands like **L2/L3**, while a well known azido derivative **L1** is considered as control throughout the study.

### Cell count assay

This assay was carried out to verify the efficiency of ligands in imparting cytotoxicity among different cell lines (2 normal and 5 tumor cell lines) in the presence of synthetic 1,2,3-triazole scaffolds at different periods of treatment (24 h and 48 h). Supporting the MTT data, the cell count assay results indicate that among the nucleoside and non-nucleoside triazole derivatives, the number of viable cells was low with nucleoside 1,2,3-triazole ligands indicating that they impart higher cytotoxicity in tumor cells. Nucleoside triazole ligands inhibited tumor cell proliferation more than the normal cell lines probably due to higher uptake of the ligands by rapidly dividing tumor cells, and their structural similarity with the natural nucleosides. Results emphasise that among the triazole ligands studied, **L3** exhibited greater inhibition of tumor cell proliferation. This was followed by **L2** and **L1**. The bar graphs shown in Figure S1 indicate that proliferation of tumor cells decreases gradually from **L1** to **L3** both in the case of 24 h and 48 h of ligand treatment. The extent of decrease of tumor cell proliferation was comparatively more with B16F10 and A549 cell lines. Details of various pharmacophores attached to 1,2,3-triazole moiety at 1^st^, 4^th^ and 5^th^ positions, are tabulated in Table S1 in Supplementary Data S1. The results obtained in cell count assays with both nucleoside and non-nucleoside synthetic triazole ligands on 7 different cell lines (both normal and tumor) are shown in Table 2.

**Table 2:** Effect of nucleoside and non-nucleoside ligands on normal and tumor human cell lines.

S.No	Ligand	Number of viable cells after 24/48 h of ligand treatment						
		CRL-2115	CRL-2120	NCM-460	He La	MCF-7	B16F10	A549
1.	L1	+/++	+/++	+/++	+/++	+/++	++/+++	+/++
2.	L2	+/++	+/++	+/++	+/++	+/++	++/+++	++/++
3.	L3	+/++	+/++	+/++	++/++	++/++	++/+++	++/+++
4.	L4	*+/++*	*+/+*	*+/++*	*+/++*	*++/++*	*++/++*	*+/++*
5.	L5	+/++	+/++	+/++	++/++	++/++	++/++	+/++
6.	L6	+/++	+/++	++/++	++/++	+/++	+/++	++/++
7.	L7	+/+	+/++	+/++	+/+	+/++	+/++	+/+
8.	L8	+/+	+/+	+/++	+/++	+/+	+/++	+/++
9.	L9	+/++	+/+	+/+	+/++	+/+	+/++	+/++
10.	L10	+/+	+/+	+/++	+/+	+/++	+/++	+/+
11.	L11	+/+	+/+	+/+	+/++	+/++	+/++	+/++
12.	L12	+/+	+/+	+/++	+/++	+/++	+/++	+/+
13.	L13	+/+	+/+	+/+	+/++	+/+	+/++	+/+
14.	L14	+/+	+/+	+/++	+/+	+/++	+/++	+/++

+ - Viable cells in the range of 80%–90% ; ++ - Viable cells in the range of 50%–60% ; +++ - Viable cells which are equal or less than 30%.

### Flow cytometry studies

**Figure 4:**
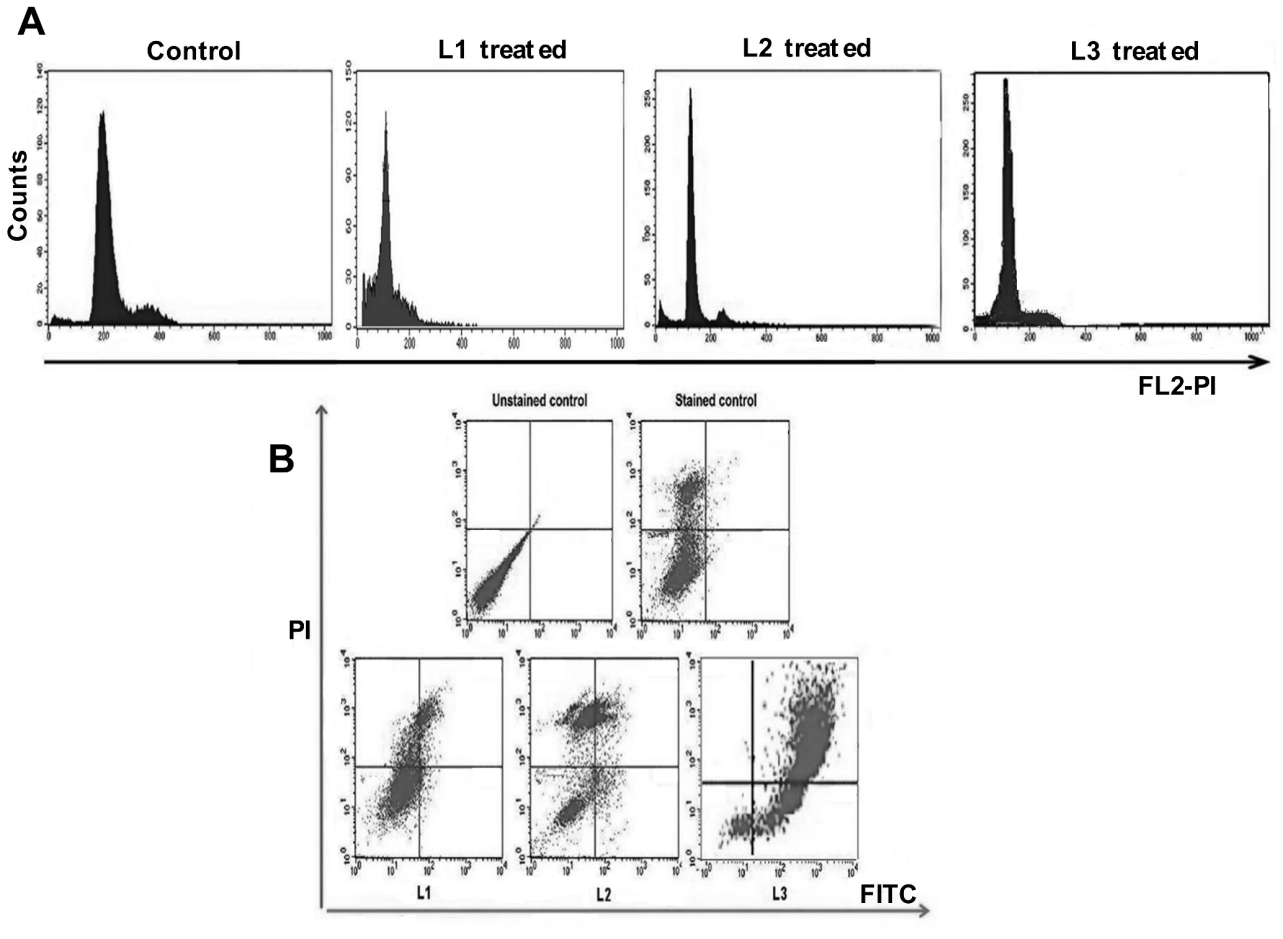
Cell cycle and dot plots obtained with three nucleoside ligands. **A.** Untreated, **L1**, **L2** and **L3** treated B16F10 cell lines were stained with Propidium Iodide (PI) and cellular DNA content frequency histograms were shown. **B.** B16F10 cells and **L1,L2** and **L3** treated cells were stained with Annexin V and Propidium Iodide and analysed for various types of cells like alive (LL), early apoptotic (LR), late apoptotic (UR) and necrotic cells (UL).

Flow cytometry experiments provide insight into the effect of ligands on various phases of cell cycle and their influence on apoptosis. Cell cycle analysis suggests that in control samples (untreated B16F10 cells), ≈60% of all cells accumulated in the G0/G1 phase, ≈24% in the S phase, and ≈8.3% in the G2/M phase. Only 1.5% of the cells are in the subG1 phase. B16F10 tumor cell lines on treatment with 50 µM of **L3**, resulted in reduction of percentage of cells in G0/G1 (4.46%), S (5.36%) and G2/M (0.91%) phases compared to untreated cells, while the percentage of cells in subG1 phase increased to 89.27%, indicating enhanced DNA fragmentation and induction of apoptosis in tumor cells. On the other hand, cells treated with even 200 µM and 125 µM of **L1** and **L2** exhibited a moderate decrease in the percentage of cells in G0/G1 (21.24% and 6.43%), S (8.36% and 8.77%) and G2/M (5.58% and 1.93%) phases, compared to untreated cells. The subG1 population in these samples increased to 61.10% and 81.5% respectively. The deconvoluted DNA histograms have been displayed in Figure 4A. The histogram depicts that the subG1 cell population has been enhanced to 10–30% with **L3** compared to other nucleoside ligands. Flow cytometry data indicates that cell cycle arrest occurs at S and G2/M phases, accompanied by an increase in the subG1 population with **L3**, indicating its potential to inhibit DNA synthesis followed by induction of apoptosis in tumor cells.

From the dot plot shown in Figure 4B, indicate that the percentage of necrotic and viable cells has reduced and the early or late apoptotic cells have increased on treating the cells with **L3**. This indicates that **L3** is more potent in inducing apoptosis in tumor cells than **L1** and **L2**. Under the same conditions, when B16F10 cells were treated with **L2**, more necrotic cells were observed compared to apoptotic cells. In **L2** treated cells, the subG1 population has increased due to the accumulation of necrotic cells rather than that of apoptotic cells. The dot blot results indicate that treatment of B16F10 cells with **L3** resulted in more apoptotic cells (81.25%) than the cells treated with **L2** (23.89%) and **L1** (14.66%).The percentage of cells accumulated in each phase of cell cycle as well as in different stages of apoptosis were shown in Tables 3 and 4 respectively. Among these phenyl-1,2,3-triazole-thymidine ligands, **L3** was found to be more ideal as it has higher capability to induce apoptosis in tumor cells.

**Table 3:** Distribution of cells in various phase of cell cycle in control and L1, L2 and L3 treated B16F10 cells.

S.No	Sample Name	Sub G1	G0/G1	S	G2/M
1	Control	01.52%	66.41%	23.81%	08.30%
2	L1	61.10%	26.94%	06.38%	05.58%
3	L2	81.52%	07.78%	08.77%	01.93%
4	L3	89.27%	04.46%	05.36%	00.91%

**Table 4:** Percentage of alive, early apoptotic, late apoptotic and necrotic cells in control and L1, L2 and L3 treated B16F10 cells.

S.No	Sample Name	Cells Alive	Early apoptotic	Late apoptotic	Necrotic	Total apoptotic cells
1	Unstained Control	99.67%	00.04%	00.27%	00.02%	00.31%
2	Stained Control	75.72%	00.46%	00.47%	23.35%	0.93%
2	L1	68.78%	02.61%	12.05%	16.56%	14.66%
3	L2	22.88%	04.82%	19.07%	53.23%	23.89%
4	L3	17.79%	20.99%	60.26%	00.96%	81.25%

### Confocal microscopy

**Figure 5:**
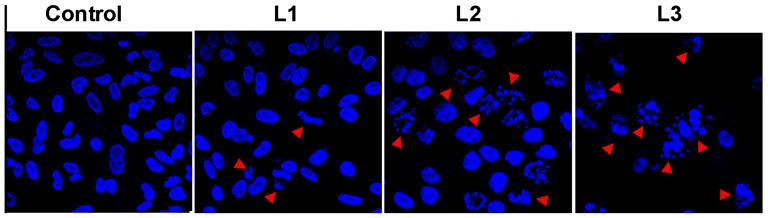
DNA fragmentation in B16F10 cells on nucleoside ligands treatment. B16F10 cells were treated with 200 µM of **L1**, 125 µM of **L2** and 50 µM of **L3** for 24 h. Control cells were treated with equal volume of 1∶1 methanol: water without ligand.

It is evident from the flow cytometry analysis that **L3** has enhanced subG1 population of cells, reduced the percentage of cells in the S phase and effectively inducing apoptosis in B16F10 cells. In order to understand the action of nucleoside 1,2,3-triazole ligands, B16F10 cell lines were treated with **L1**, **L2**, **L3** at their respective half maximal inhibitory concentration (i.e. 200 µM, 125 µM and 50 µM) for 24 h period. Examination of ligand treated cells under the fluorescence microscope indicated higher nuclear condensation and DNA fragmentation in nucleoside triazole ligand treated tumor cells than in untreated cells. The cell surface was altered or shrunken, DNA fragmented was enhanced and the number of apoptotic cells was increased in cells treated with **L3** compared to **L1**/**L2**. Figure 5 shows the confocal microscopy results with B16F10 cell lines on treatment with nucleoside triazole ligands. Flow cytometry and confocal microscopy results shows that among the nucleoside ligands tested, induction of apoptosis in tumor cells and fragmentation was highest in the presence of **L3** followed by **L2** and **L1**.

### Preclinical studies

**Figure 6:**
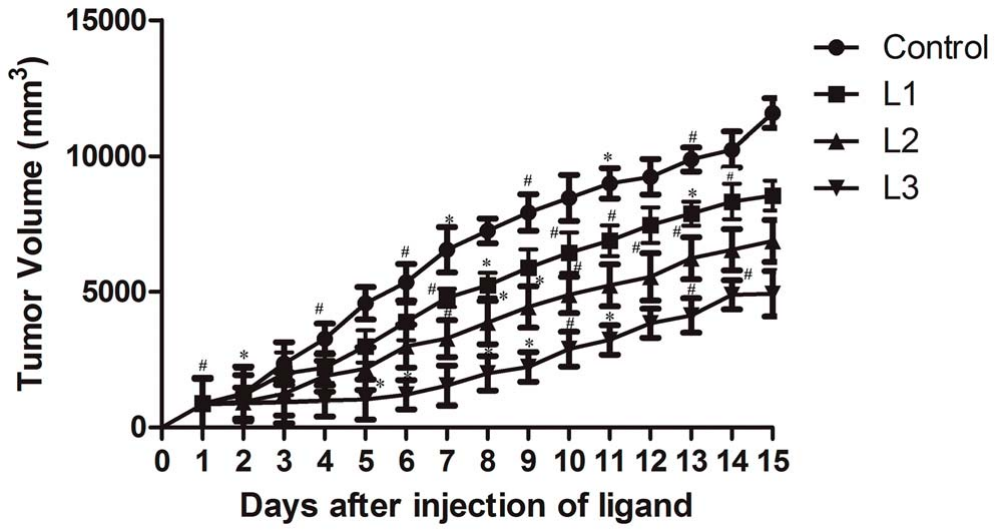
Varation of induced tumor size on treatment with nucleoside ligands. Tumor was induced in C57BL6/J mice by injecting B16F10 cells subcutaneously and treated with 200 µM of **L1**, 125 µM of **L2** and 50 µM of **L3** and an equal volume of 1∶1 methanol: water (without ligand) was treated as vehicle control. Group treated with an equal volume of 1∶1 methanol: water, vehicle control (4 mice) marked •. Group treated with **L1**, **L2** and **L3** (4 mice each) were marked ▪, ▴ and ▾ respectively. Tumor volumes were measured at fixed time points and expressed in mm^3^. Each experiment was repeated thrice and the mean values were plotted. The set of data with p value≤0.001 and 0.01 were marked with * and # respectively. The statistical data with p value≤0.05 was left unmarked.

Experiments such as flow cytometry, confocal microscopy and MTT assay with normal and tumor cell lines indicate that **L3** has the potential to terminate cell cycle at S phase, increase subG1 population, enhance DNA fragmentation and induce apoptosis in tumor cells at comparatively lower concentration (50 µM). However, the effect of synthetic triazole ligands under physiological conditions (under *in vivo* situation) provides unfeigned data on the actual potential of ligands as anti-proliferative agents. Therefore, it would be interesting to observe variations in tumor sizes among tumor-induced mice after ligand treatment. Figure 6 indicates that in the group of mice treated with **L1/L2**, the tumor size increased to 6000–8000 mm^3^, whereas in the group of mice treated with **L3**, the size of tumor remained at ≈4500 mm^3^. Thus, *in vivo* data also support earlier experimental results and indicate that **L3** has better tumor controlling capability than **L1/L2**.

Further, the survival of mice treated with different ligands was checked and compared with the control group. The details of survival data obtained from 4 different sets of mice are tabulated in Table S2 in Supplementary Data S1. Among the mice treated with nucleoside ligands, the mice treated with **L3** survived for longer period (about 6 weeks) indicating that **L3** was efficient in controlling tumor cell proliferation without developing harmful effects in tumor-induced mice. Untreated mice, **L1** and **L2** treated mice died earlier (approximately in 3, 4 and 5 weeks, respectively). Experiments to understand the reason for higher longevity of mice with **L3** are in progress.

### Stop assay or Inhibition of *In vitro* DNA synthesis

It is already known that 3′ modified nucleosides inhibit DNA synthesis. Earlier, 3′-Phenyl-1,2,3-triazole-thymidine and similar kind of triazole nucleoside ligands were reported to terminate DNA synthesis in tumor cell lines [46]. In the present study, flow cytometry and confocal studies indicate that the cell cycle was inhibited at S phase and DNA fragmentation occurred on treatment with nucleoside 1,2,3-triazole ligands. Higher the efficiency of inhibition of DNA synthesis, greater will be the effect of the ligand on DNA fragmentation.

**Figure 7:**
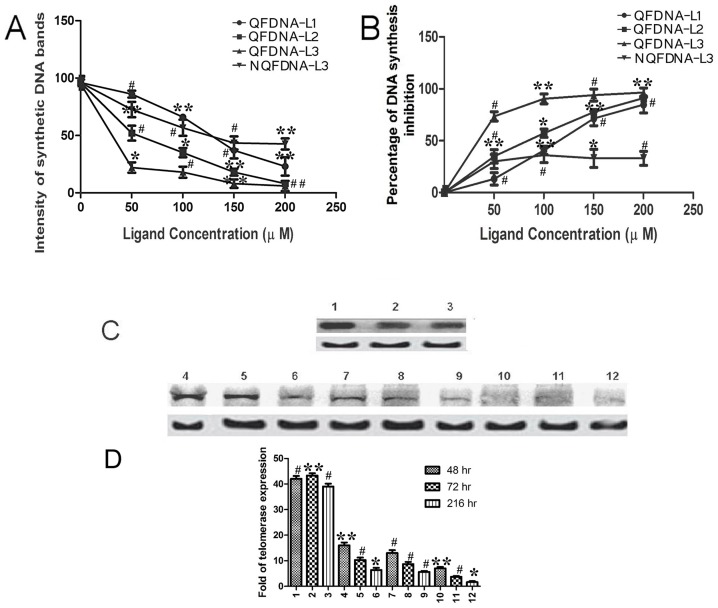
Effect of ligands on *in vitro* DNA synthesis inhibition and telomerase expression. **A.** Line graph indicating the variation in band intensity with **L1**, **L2** and **L3** ligands at various concentrations using QF DNA and **L3** alone with NQF DNA. **B.** Percentage of DNA synthesis inhibition by **L1**, **L2** and **L3** with QF DNA and **L3** alone with NQF DNA. **C.** Western blot showing the TERT and β-actin bands. Lane 1,2,3 corresponds to 48 h, 72 h and 216 h of treatment with vehicle control (control samples); lane 4,5,6, lane 7,8,9 and lane 10,11,12 corresponds to 48 h, 72 h and 216 h of **L1**, **L2** and **L3** treatment respectively. Top and bottom bands correspond to TERT and β-actin respectively. **D.** Bar graph showing the comparative intensities of each band in western blot. The numbers marked on the x-axis represents the number of sample in western blot. The set of data with reducing order of significance (p value≤0.001, 0.01 and 0.05) were marked with *, # and ** respectively.

The role of nucleoside triazole ligands in inhibiting DNA synthesis by QF DNA and NQF DNA was studied by using the protocol reported earlier [47], [48]. Inhibition of DNA synthesis was studied using 5 µM QF DNA and nucleoside triazole ligands (**L1, L2, L3**) at different concentrations (50 µM, 100 µM, 150 µM, 200 µM). It was found that the inhibition of DNA synthesis with QF DNA was considerably high (around 80–90%). The *in vitro* DNA synthesis products obtained with QF DNA and **L1, L2, L3** were resolved by loading on 12% nondenaturing polyacrylamide gel as shown in Figure S2. The results reveal that **L3** completely inhibited DNA synthesis in the concentration range of 50–100 µM, whereas with **L1** and **L2**, maximum inhibition was observed at higher ligand concentrations (150–200 µM). The results matched with the data provided by MTT assay. However, inhibition of *in vitro* DNA synthesis with NQF DNA was studied only with **L3** ligand, because it was identified as the most potential anti-proliferative ligand among the triazole scaffolds considered in the present study. Competitive inhibition of DNA synthesis by **L3** ligand in the presence of natural dNTPs and NQF DNA is shown in Figure S2A. With NQF DNA, inhibition of *in vitro* DNA synthesis by **L3** was moderate (around 33%). From Figure 7A, it is evident that the intensities of synthetic DNA bands were less with **L2/L3** than **L1**, indicating Phenyl triazole ligands were the better DNA synthesis inhibitors than azido scaffolds. The competitive inhibition by various nucleoside triazole ligands and the inhibition constants (K_i_) for each of the ligands was calculated using Cheng-Prusoff equation (equation-2, shown under experimental data). They were found to be 2.94 (±0.091) µM, 1.84 (±0.079) µM and 0.735 (±0.087) µM for **L1**, **L2** and **L3**, respectively. The inhibition constant with **L3** was lowest among those tested, indicating its ease to get incorporated into the growing DNA chain.

The results obtained from the stop assay indicate that quadruplex complex formation and stabilization of quadruplex complex by suitable ligands would enhance inhibition of DNA synthesis. With NQF DNA, about 33% inhibition was observed, indicating misincorporation of **L3** into the growing DNA strand alone was causing inhibition of DNA synthesis. The misincorporation of modified nucleosides probably plays a primary role in terminating the DNA synthesis. However, the inhibition of DNA synthesis was less with NQF DNA compared to QF DNA, emphasising the role of quadruplex formation in DNA synthesis inhibition. Intensity of synthesized DNA bands obtained with different ligands using QF and NQF DNA are shown in Figure S2. The percentage of DNA synthesis inhibition by **L1**, **L2** and **L3** with QF DNA at the respective half maximal inhibitory concentration was calculated using equation-1 (shown under experimental section) and was found to be 84%, 91% and 94%, respectively. Percentage of DNA synthesis inhibition with QF and NQF DNA is graphically shown in Figure 7B. Figure S3 illustrates the proposed mechanism by which **L3** terminates DNA synthesis in NQF DNA and QF DNA.

### Western blot

Telomerase has been reported to play an important role in tumorigenesis [49]. Overproduction of telomerase has been observed in more than 85% of tumor cells than in normal cells [50]. It was reported earlier that an efficient telomerase inhibitor such as 3′-azido-3′-deoxythymidine (**L1**) [51]–[55] induces apoptosis in tumor cells. To find out the effect of phenyl-triazole ligand treatment on the level of telomerase expression in the tissues of tumor-induced mice, western blot assay was performed. Western blot results show that among the nucleotide triazole ligands, level of telomerase expression was low in tumor tissues treated with **L3** than in **L1** and **L2**. Figure 7C shows that telomerase expression levels were higher in control tissue samples (untreated tumor tissue), than in the ligand treated ones. We speculate that the reduction of telomerase expression was due to continuous inhibition of telomerase activity by nucleoside ligands. Levels of telomerase expression reduced gradually in the tumor tissues treated with nucleoside ligands from 48 h to 216 h of treatment (as shown in Figure 7D). Percentages of reduction of telomerase expression on 216 h of treatment with **L1**, **L2** and **L3** were calculated using the equation 1, (under materials and methods section) and they were found to be 80%, 88% and 95%, respectively.

### Binding and interaction studies

Stop assay results show that stabilization of quadruplex DNA and the inhibition of DNA synthesis were related to each other and it has some role to play in induction of apoptosis. G-quadruplex DNA interaction with 3′ modified nucleoside ligands would throw some light on the nature of DNA-ligand interactions, their potential to stabilize G-quadruplex DNA and role of ligand structure in quadruplex DNA-ligand interaction. ITC and melting studies were carried out to investigate the efficiency of ligands interaction, mode of binding and affinity of ligands towards human telomere DNA (HQF DNA).

**Figure 8:**
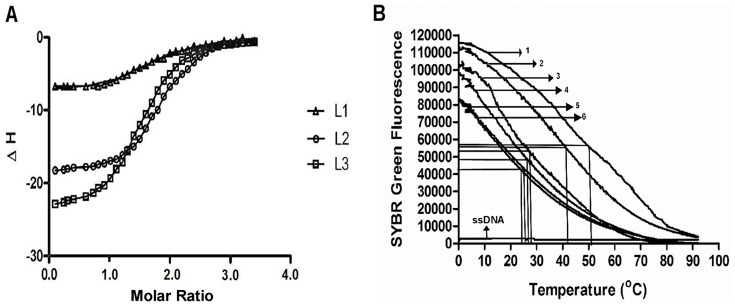
Microcalorimetric and DNA melting studies in presence of nucleoside ligands. **A.** ITC binding isotherms obtained when **L1**, **L2** and **L3** ligands were titrated with HQF DNA in 100 mM KBPES (pH 7.0) buffer. **B.** Melting profile of DNA with various ligands. HQF DNA +**L3** (marked-1); HQF DNA+**L2** (marked 2); HQF DNA+ **L1** - (marked 3) ; HQF DNA alone - (marked 4); dsDNA+ **L3** ( marked 5); dsDNA alone (marked 6) and ssDNA (poly-dT alone).

Isothermal titration calorimetry is a sensitive microcalorimetric technique for determining binding affinity, stoichiometry and thermodynamic parameters of quadruplex DNA-ligand complex [56]. Interaction between HQF DNA (10 µM) with the nucleoside ligands (60 µM) was performed at 25°C in 100 mM KBPES (pH 7.0) buffer and the data obtained are shown in Table S3 in Supplementary Data S1. On integrating the heats produced per injection, with respect to time and conversion to per mole basis, the binding isotherm (Figure 8A) corresponding to each ligand was obtained. Figure 8A indicates that two ligand molecules would interact with each molecule of quadruplex DNA. After making blank correction, the heat data was fitted with “two-independent sites” model and analyzed using Origin 7.1 to calculate thermodynamic parameters.

The microcalorimetry results indicate that the interaction of ligand takes place in two different modes : one with higher affinity (mode-1, preferred interaction) and the other with lower affinity (mode-2, less preferred interaction). We propose that the higher affinity interaction (mode-1), may be due to the ligand binding externally to quadruplex DNA. The mode-1 interaction occurs with higher binding constant (K_d_≈10^−7^ M) and with more favourable entropy change. The low affinity interaction (mode-2) is due to either π-π stacking interaction of nucleoside triazole ligands with guanines of quadruplex DNA or due to binding of ligands to adenosine bases in the loop (end loop binding with binding constant in the range of K_d_≈10^−5^ M), which occurs with more favourable enthalpy change [57].

### Melting studies

In the present study, SYBR Green fluorescence was monitored to find melting of ligand-DNA complexes and to know the efficacy of each triazole ligand in stabilizing the quadruplex complex. Among the different synthetic ligands, the T_m_ of HQF DNA was 52°C with **L3**, 42°C with **L2** and 28°C with **L1**. T_m_ of dsDNA alone, dsDNA with **L3**, HQF DNA alone and HQF DNA with other non-nucleoside triazole scaffolds (**L4–L14**) were observed to be 24°C, 25°C, 27°C and 27–33°C, respectively. SYBR Green fluorescence was very low with single stranded DNA, because SYBR Green has less binding to ssDNA. T_m_ of dsDNA does not alter much in the presence or absence of **L3**, suggesting that **L3** has minimal interaction with dsDNA. These results indicate that **L3** specifically interacts with quadruplex DNA and stabilizes the complex. It is interesting to note that attaching an electronegative moiety to the phenyl group of **L2** ligand, enhance the stability of quadruplex DNA. From the present study, we clearly demonstrate that quadruplex DNA stabilizers act as efficient inhibitors of DNA synthesis, telomerase expression and act as pro-apoptotic ligands. It was shown earlier that ligands that stabilize quadruplex DNA inhibit telomerase expression [58], [59] and function as antitumor agents.

From the melting assay results, the order of HQF DNA stabilization by triazole and azido ligands is as follows: **L3>L2>(L4–L14)>L1**. Results indicate that size and shape of the interacting ligand is crucial for efficient stabilization of quadruplex DNA. It was shown earlier that ligands such as TMPyP4 provide higher stability to quadruplex DNA because its molecular shape and size is similar to that of guanine tetrad (diameter of quadruplex DNA complex is ≈13°A) [60], [61]. Previous studies on quadruplex DNA–ligand interactions indicate that molecules having molecular weight in the range of 2000–5000 Da provide better stability to quadruplex DNA [62]. The non-nucleoside ligands **L4–L14** fail to stabilize quadruplex DNA probably because of their smaller size or weaker interaction with quadruplex DNA. Though the IC_50_ values were not significant, studies on nucleoside ligands such as **L1**, **L2** and **L3** are useful in building new molecules with improved pro-apoptotic activity, enhance stability of quadruplex DNA, effective inhibition of DNA synthesis. The details of structure of both nucleoside and non-nucleoside ligands, melting temperature of HQF DNA in presence of **L1**, **L2**, **L3** and **L4**–**L14** and their IUPAC names are provided in Table S4 in Supplementary Data S1 and the melting profiles of HQF DNA with **L1**, **L2**, **L3**, HQF DNA alone, dsDNA, dsDNA with **L3**, ssDNA are shown in Figure 8B.

By and large, from the results obtained from the present study, the following aspects were deduced about the potential 1,2,3-triazole ligand namely, 3′-Chrolophenyl-1,2,3-triazole-thymidine:

1. The nucleoside triazole scaffolds like 3′-Phenyl-1,2,3-triazole-thymidine and 3′-4-Chloro-phenyl- 1,2,3-triazole-thymidine ligands (**L2** and **L3**) exhibited higher selectivity and greater stability to quadruplex DNA than the 3′-azidothymidine (**L1**) and non-nucleoside triazole scaffolds (**L4–L14**).
2. Termination of *in vitro* DNA synthesis by 3′ modified nucleosides is primarily due to its misincorporation into the growing DNA strand. Termination was effective in QF DNA (about 3 times more effective) than in NQF DNA, indicating the role of formation and stabilization of quadruplex DNA complex in the inhibition of DNA synthesis. Higher the quadruplex DNA stabilization, more efficient would be the inhibition of DNA synthesis.
3. **L3** inhibits *in vitro* DNA synthesis more efficiently than the other two nucleoside triazole ligands. The inhibition constant (K_i_) was found to be the lowest (0.735 µM) among the nucleoside triazole ligands used in the present study, indicating higher affinity of **L3** towards polymerase enzyme and ease to get incorporated into the growing DNA strand.
4. **L3** reduces telomerase levels at lower concentration (50 µM) than **L1/L2**. **L1** was reported to inhibit telomerase at concentration around 200 µM [63].
5. DNA fragmentation, inhibition of *in vitro* DNA synthesis and induction of apoptosis was more with 3′-4-Chlorophenyl-1,2,3-triazole-thymidine (**L3**) than the 3′-Phenyl-1,2,3-triazole-thymidine (**L2**). This shows that attaching an electronegative element to the phenyl group, improves the pro-apoptotic activity of the ligand and function as better substrate for enzymatic reactions. Similar observation was made earlier with 3′-Flurophenyl-1,2,3-triazole-thymidine [64].

## Conclusion

From the present study, we report that among the library of 1,2,3-triazole ligands, nucleoside ligands are more efficient than non-nucleoside ligands in controlling tumor cell proliferation. Among the nucleoside ligands, 4-Chlorophenyl-1,2,3-trizole-thymidine (**L3**) exhibits higher potential in bringing down tumor cell proliferation. **L3** enhances subG1 population of cells, DNA fragmentation and induces apoptosis in tumor cells. Among the nucleoside 1,2,3-triazole ligands, **L3** has profound effect on tumor size reduction, enhancing longevity and lowering telomerase levels in tumor-induced mice. It is interesting to observe that **L3** specifically interact and effectively stabilize quadruplex DNA. Stop assay results indicate that quadruplex DNA structure stabilization by synthetic ligands will effectively inhibit *in vitro* DNA synthesis. **L3** binds externally to HQF DNA and stoichiometry of quadruplex DNA: nucleoside ligand is 1∶2. Even though the IC_50_ values of Phenyl-1,2,3-triazole-thymidine molecules (**L2** and **L3**) are not significantly low to consider them as a candidate molecules for therapeutic purposes, the results from the present study are useful in understanding the role of pharmacorphores on pro-apoptotic activity of ligands, for building suitable molecules to improve quadruplex DNA stability, inhibit *in vitro* DNA synthesis and reduce expression of telomerase in tumor cells.

## Supporting Information

Figure S1 **Cell count assay using normal and tumor cell lines.** Cell count assay results with normal as well as tumor cell lines after 24 h and 48 h of treatment with nucleoside ligands (**L1**, **L2** and **L3**). Experiment was repeated thrice and the mean values were plotted. The set of data with reducing order of significance (p value≤0.001, 0.01 and 0.05) were marked with *, # and ** respectively.
(TIF)

Figure S2 **Inhibition of** ***in vitro*** **DNA synthesis with NQF and QF DNA in presence of nucleoside ligands.** **A.** Stop assay with **L3** and NQF DNA: Lane 1–5 show 5 µM NQF DNA with 0 µM, 50 µM,100 µM,150 µM,200 µM of **L3.** **Figure 7** **B,C,D.** Stop assay with **L1**, **L2** and **L3** and QF DNA: Lane 1–5 show 5 µM QF DNA with 0 µM, 50 µM, 100 µM, 150 µM, 200 µM of **L1, L2 and L3** respectively. The control lane for QF DNA is considered common for B,C and D figures.
(TIF)

Figure S3 **Possible mechanism of termination of DNA synthesis with NQF and QF DNA in presence of L3.** Schematic representation of mechanism of termination of DNA synthesis followed by DNA fragmentation and induction of apoptosis with QF and NQF DNA in presence of L3 and dNTPs.
(TIF)

Supplementary Data S1 **Contains Tables S1, S2, S3, S4.**
(DOCX)
