# Supplementary figures and images for: Phenyl 1,2,3-Triazole-Thymidine Ligands Stabilize G-Quadruplex DNA, Inhibit DNA Synthesis and Potentially Reduce Tumor Cell Proliferation over 3′-Azido Deoxythymidine
Source: PLoS One. 2013 Aug 19;8(8):e70798. doi: 10.1371/journal.pone.0070798 (PMC3747139; doi:10.1371/journal.pone.0070798)

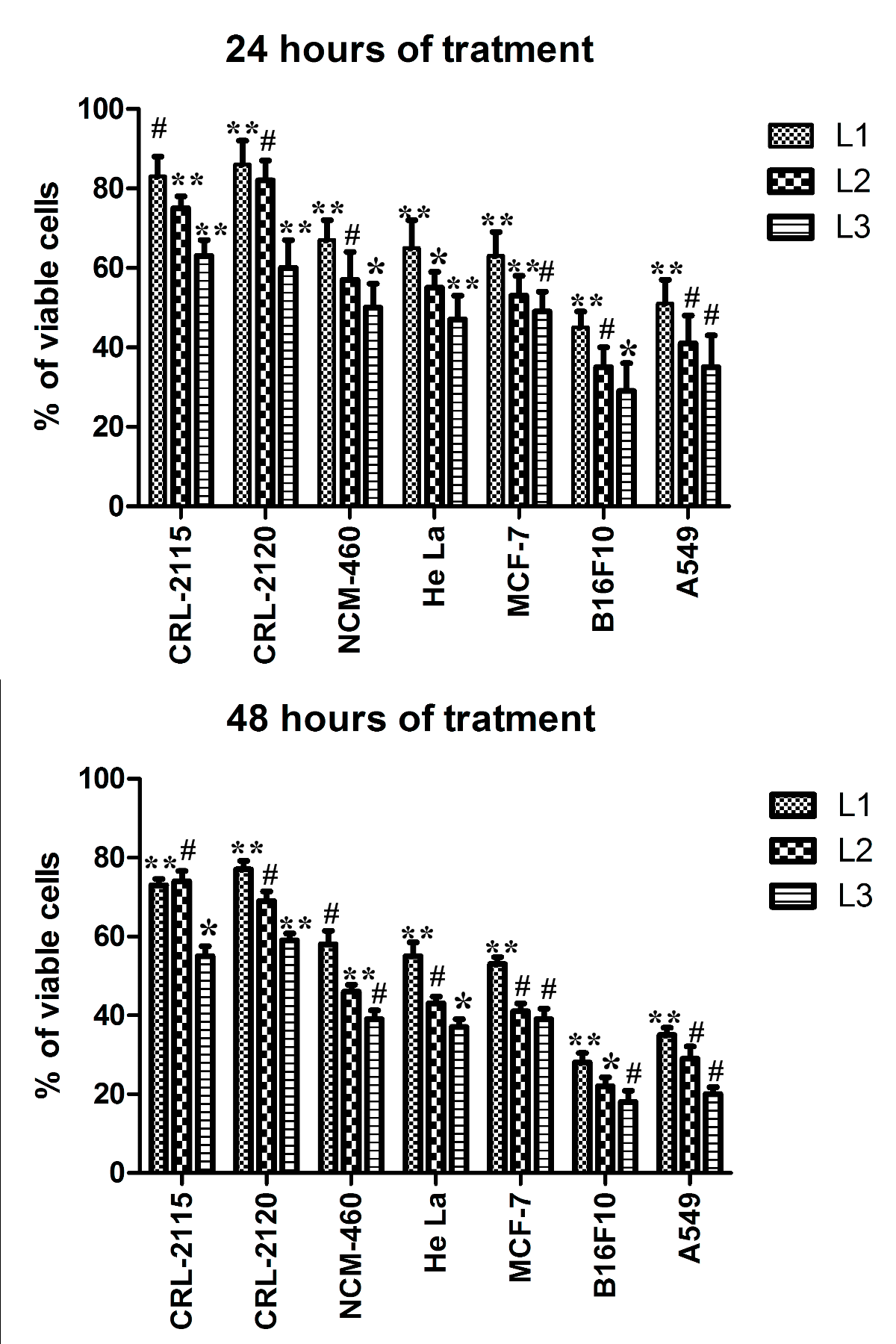

Supplement: Figure S1 — Cell count assay using normal and tumor cell lines. Cell count assay results with normal as well as tumor cell lines after 24 h and 48 h of treatment with nucleoside ligands (L1, L2 and L3). Experiment was repeated thrice and the mean values were plotted. The set of data with reducing order of significance (p value≤0.001, 0.01 and 0.05) were marked with *, # and ** respectively. (TIF) [file pone.0070798.s001.tif]

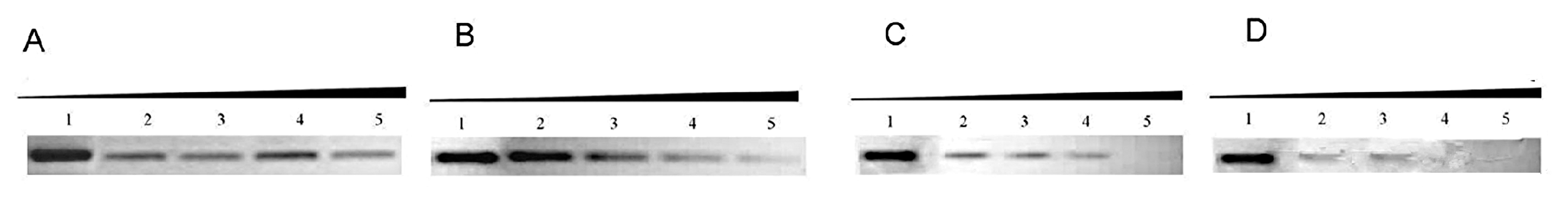

Supplement: Figure S2 — Inhibition of in vitro DNA synthesis with NQF and QF DNA in presence of nucleoside ligands. A. Stop assay with L3 and NQF DNA: Lane 1–5 show 5 µM NQF DNA with 0 µM, 50 µM,100 µM,150 µM,200 µM of L3. Figure 7 B,C,D. Stop assay with L1, L2 and L3 and QF DNA: Lane 1–5 show 5 µM QF DNA with 0 µM, 50 µM, 100 µM, 150 µM, 200 µM of L1, L2 and L3 respectively. The control lane for QF DNA is considered common for B,C and D figures. (TIF) [file pone.0070798.s002.tif]

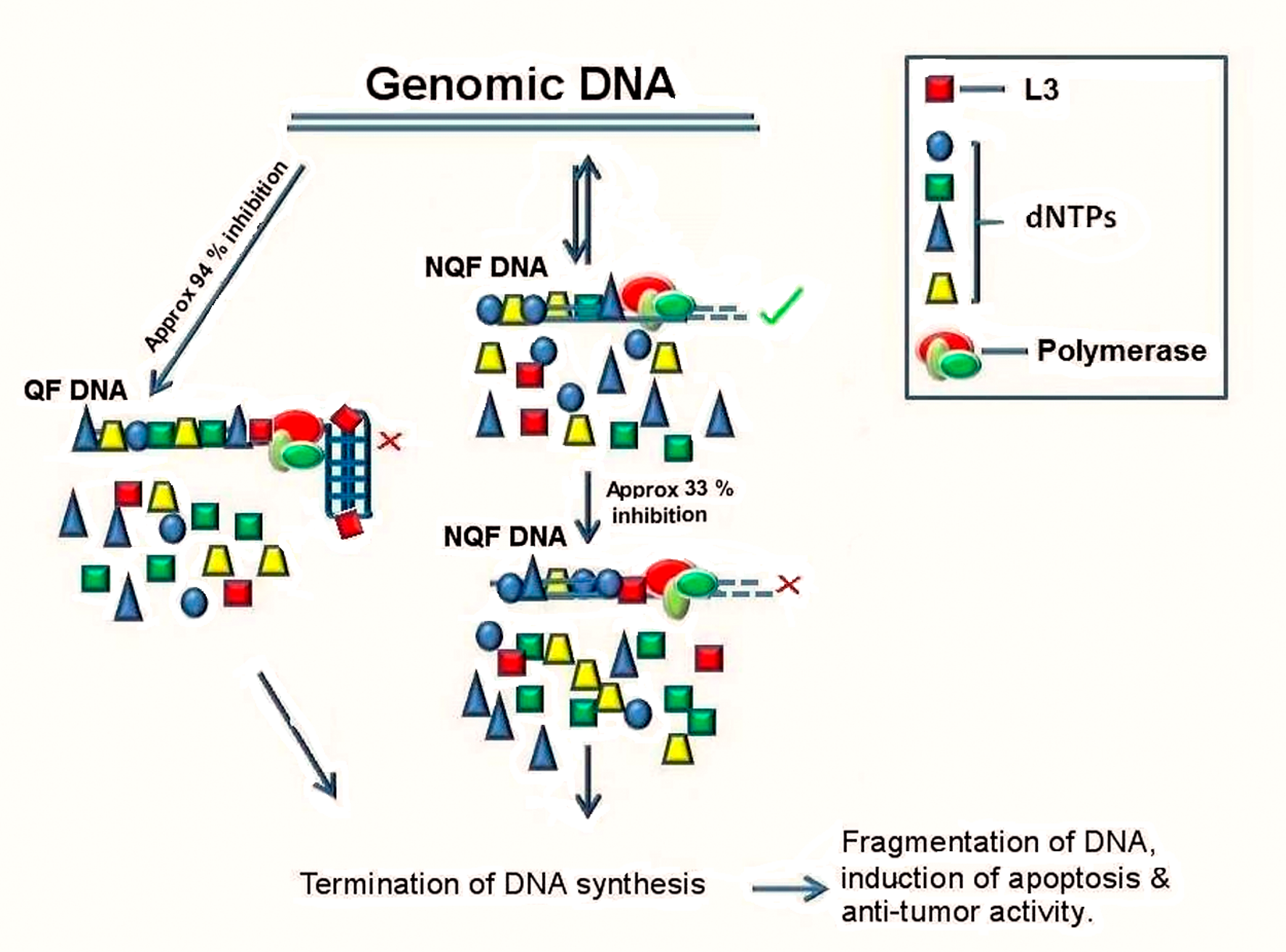

Supplement: Figure S3 — Possible mechanism of termination of DNA synthesis with NQF and QF DNA in presence of L3. Schematic representation of mechanism of termination of DNA synthesis followed by DNA fragmentation and induction of apoptosis with QF and NQF DNA in presence of L3 and dNTPs. (TIF) [file pone.0070798.s003.tif]
