# Supplementary material for: Phenyl 1,2,3-Triazole-Thymidine Ligands Stabilize G-Quadruplex DNA, Inhibit DNA Synthesis and Potentially Reduce Tumor Cell Proliferation over 3′-Azido Deoxythymidine
Source: PLoS One. 2013 Aug 19;8(8):e70798. doi: 10.1371/journal.pone.0070798 (PMC3747139; doi:10.1371/journal.pone.0070798)
Supplement: Supplementary Data S1 — Contains Tables S1, S2, S3, S4. (DOCX) [file pone.0070798.s004.docx]

**SUPPORTING INFORMATION**

**Table S1.** Details of 1,2,3-triazole derivatives used in the present study and the structure of

various pharmacophores attached to the 1,2,3-triazole ring. **L1** is not included in

the list because it does not contain triazole ring.


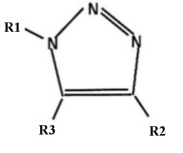


| Compound No | -R1 | -R2 | -R3 |
| --- | --- | --- | --- |
| L2 | **** | 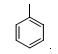 | H |
| L3 | **** | 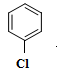 | H |
| L4 | **** |  | **** |
| L5 | **** | **** | **** |
| L6 | **** | **** | **** |
| L7 | **** | **** | **** |
| L8 | **** | **** | **** |
| L9 | **** | COOEt | **** |
| L10 | **** | COOEt | **** |
| L11 | **** | COOEt | **** |
| L12 | **** | **** | **** |
| L13 | **** | **** | **** |
| L14 | **** | **** | **** |

**Table.S2.** Survival details of tumor-induced mice after nucleoside 1,2,3-triazole thymidine

ligands (**L1**, **L2** and **L3**) treatment

| **S.No** | **Ligand name** | **Number of days survived** |
| --- | --- | --- |
| 1 | Control | 21 (±4) days |
| 2 | L1 | 25 (±5) days |
| 3 | L2 | 31 (±4) days |
| 4 | L3 | 40 (±6) days |

**Table S3.** ITC data obtained when nucleoside ligands interact with quadruplex DNA

complex formed by human telomeric DNA, d(TTAGGG)_4._

| **ITC Derived Thermodynamic Parameters** | **L1-Quadruplex DNA** | **L2-Quadruplex DNA** | **L3- Quadruplex DNA** |
| --- | --- | --- | --- |
| K1 x 10^-7^ | 0.72 (± 0.3) | 1.35 (± 0.1) | 2.45 (± 0.4) |
| ΔG1 (kcal/mol) | -9.16 | - 8.93 | - 6.94 |
| ΔH1 (kcal/mol) | - 0.91 (± 0.2) | - 1.72 (± 0.1) | - 2.16 (± 0.1) |
| -TΔS1 (kcal /mol ) | -8.25 | - 7.21 | - 4.79 |
| K2 x 10^-5^ | 2.14 (± 0.2) | 2.61 (± 0.3) | 2.82 (±0.5) |
| ΔG2 (kcal/mol) | -8.11 | -8.28 | -8.41 |
| ΔH2 (kcal/mol) | - 7.12 ( ± 0.2) | - 6.86 (± 0.1) | - 4.54 (± 0.1) |
| -TΔS2 (kcal /mol) | - 0.97 | - 1.42 | - 3.87 |

Parameters mentioned above are for a two-sites binding model. The uncertainties mentioned for the two ﬁtting parameters, Ki and ∆Hi, were determined from Monte Carlo analysis.

**Table S4.** Structure, IUPAC name, melting temperature and molecular weight of azido and

synthetic 1,2,3-triazole ligands used in the present study.

| **Ligand Name** | **Structure** | **Name** | **T_m_  (^o^ C)** | **Mol. weight** |
| --- | --- | --- | --- | --- |
| L1 |  | 1- (4-azido 5- (hydroxyl methyl)tetrahydrofuran-2-yl)-5-methyl pyrimidi-ne-2,4(*1H*,*3H*)-dione. | 28 | 267.2 |
| L2. |  | 1-((2R,4S,5S)-5-(Hydroxymethyl)-4-(4-phenyl)-1H-1,2,3-triazol-1-yl)tetrahydrofuran-2-yl)-5-nethylpyrimidine-2,4(1H,3H)-dione. | 42 | 369.4 |
| L3 | 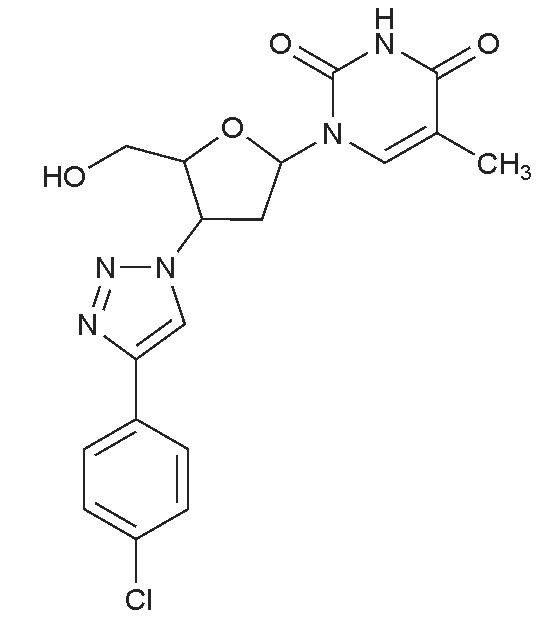 | 1-(4-(4-(4-chlorophenly)-1H-1,2,3-triazole-1-yl)-5-(hydroxymethyl)tetrahydrofuran-2-yl)-5-methylpyrimidine-2,3(1H,3H)-dione | 52 | 404.9 |
| L4 |  | 1-Benzyl-4,5-diphenyl-1H-1,2,3-triazole | 32 | 311.4 |
| L5 |  | (E)-1-Benzyl-4-butyl-5-styryl-1H-1,2,3-triazole | 33 | 317.2 |
| L6 |  | 3-(3-Benzyl-5-butyl-3H-1,2,3-triazol-4-yl)phenol | 33 | 307.2 |
| L7 |  | 4,5-Diphenyl-1-((tetrahydro-2H-pyran-2-yl)methyl)-1H-1,2,3-triazole | 29 | 319.2 |
| L8 |  | 3-(4-Phenyl-1-(tetrahydro-2H-pyran-2-yl)methyl)-1H-1,2,3-triazol-5yl)phenol | 27 | 335.2 |
| L9 |  | Ethyl 1-benzyl-5-phenyl-1H-1,2,3-triazole-4-carboxylate | 29 | 307.1 |
| L10 |  | (E)-Ethyl 1-benzyl-5-styryl-1H-1,2,3-triazole-4-carboxylate | 33 | 333.2 |
| L11 |  | Ethyl 1-benzyl-5-(3-hydroxyphenyl)-1H-1,2,3-triazole-4-carboxylate | 28 | 323.1 |
| L12 |  | (*E*)-4-Phenyl-5-styryl-1-((tetrahydro-*2H*-pyran-2-yl)methyl)-*1H*-1,2,3-triazole | 31 | 345.2 |
| L13 |  | 3-(5-Phenyl-3-((tetrahydro-*2H*-pyran-2-yl)methyl)-*3H*-1,2,3-triazol-4-yl)phenol | 33 | 335.4 |
| L14 |  | 5-(4-Methoxyphenyl)-4-phenyl-1-((tetrahydro-*2H*-pyran-2-yl)methyl)-*1H*-1,2,3-triazole | 27 | 349.2 |
